# Flexible Risk Evidence Combination Rules in Breast Cancer Precision Therapy

**DOI:** 10.3390/jpm13010119

**Published:** 2023-01-05

**Authors:** Michael Kenn, Rudolf Karch, Christian F. Singer, Georg Dorffner, Wolfgang Schreiner

**Affiliations:** 1 Section of Biosimulation and Bioinformatics, Center for Medical Statistics, Informatics and Intelligent Systems (CeMSIIS), Medical University of Vienna, Spitalgasse 23, 1090 Vienna, Austria; 2 Section for Artificial Intelligence, Center for Medical Data Science, Medical University of Vienna, Währingerstraße 25a, 1090 Vienna, Austria; 3 Section for Medical Statistics, Center for Medical Data Science, Medical University of Vienna, Währingerstraße 25a, 1090 Vienna, Austria; 4 Translational Gynecology Group, Department of Obstetrics and Gynecology Comprehensive Cancer Center, Medical University of Vienna, Waehringer Guertel 18-20, 1090 Vienna, Austria

**Keywords:** evidence theory, theory of belief functions, Dempster-Shafer theory, breast cancer, hormone receptor status, precision medicine, personalized medicine, data science, mathematical oncology

## Abstract

Evidence theory by Dempster-Shafer for determination of hormone receptor status in breast cancer samples was introduced in our previous paper. One major topic pointed out here is the link between pieces of evidence found from different origins. In this paper the challenge of selecting appropriate ways of fusing evidence, depending on the type and quality of data involved is addressed. A parameterized family of evidence combination rules, covering the full range of potential needs, from emphasizing discrepancies in the measurements to aspiring accordance, is covered. The consequences for real patient samples are shown by modeling different decision strategies.

## 1. Introduction

The Dempster-Shafer theory of evidence (DST) is a generalized framework in probability theory. First introduced by Dempster between 1966 [1] and 1968 [2] in the context of Bayesian inference [3], Shafer perpetuated his ideas into a comprehensive theory in a book in 1976 [4]. A short summary of DST with an illustrative example of how to create and combine pieces of evidence was given in 1986 by Zadeh [5].

In 1988 Smets [6] (Chapter 9) framed the concept of credibility in terms of mathematical logic. In contrary to Shafer [4] he propagated the “open-world assumption”, thus the possibility of outcomes beyond the “frame of discernment” (e.g., example of broken coin). At the same time, in 1988, Dubois and Prade [7] gave an axiomatic description of how to define and combine pieces of evidence mathematically.

One common approach to DST is via the “transferable belief model” (TBM), which Smets introduced in 1990 [8]. In the TBM evidence is fully described by “basic belief masses” (BBM). Sometimes the BBM is alternatively called “basic belief assignment” (BBA) [9]. The open world-assumption is achieved by assuming a positive value for the BBM of the empty set, as discussed in 1992 [10]. Conditioned belief and plausibility were embedded into a generalized Bayesian theorem in 1993 [11]. A procedure for a two-step decision making process within the TBM was outlined in 1994 [12]. In the first step evidence is based on belief functions as defined in DST and is called “credal” level. The following is a reduction to general probability functions, which are then used for decision making. This step is called “pignistic” level.

Among others, an important elaboration of DST is given by the “Theory of Hints”, which was outlined by Kohlas in 1995 [13]. In 1991, Gebhardt [14] introduced the “context model” to distinguish between vagueness and uncertainty which also covers topics such as refinement and coarsening. The “Dezert-Smarandache theory” (DSmT) [15] specifically targets the problem of imprecise, uncertain, and highly conflicting sources of data for information fusion.

While in probability theory the calculus with probabilities is inherent, in DST the exertion of influence between pieces of evidence opens a wide field of facilities. Combining two pieces of evidence to improved evidence is, in general, accomplished by evidence combination rules (ECR). There is certainly a large variety of meaningful ECRs. Dempster’s original suggestion, which distributes inconsistent BBMs equally among others, was the most obvious Dempster ECR [4]. The rule is commutative and associative, but fails when sources of evidence become incompatible or conflicting. To overcome the problem of combining strongly contradicting pieces of evidence, Yager 1987 [16] suggested assigning inconsistent BBMs to the BBM of total ignorance. A good summary of about ten popular ECRs is given therein [17,18]. More sophisticated are “Proportional Conflict Redistribution” rules (PCR) [19,20] within DSmT or some of the improvements made [21].

As an alternative to Dempster’s ECR and to overcoming the problem of conflicting evidence, Shafer suggested [18] to manipulate mass functions by weighting and discounting (“belief in belief”) rather than to diversify the ECR itself. In DSmT [15] evidence from several origins can additionally be weighted by importance.

The TBM gives a procedure of how to convert evidence into probabilities. However, there is a push for decision making based on evidence. An axiomatic approach was given in 1990 [22]. The inclusion of loss functions for classification were discussed in 1997 [9]. A review of decision-making strategies based on the theory of Neumann and Morgenstern from 1943 [23] (60th anniversary reprint [24]) was given in 2019 in Denœux [25].

There are many use cases for DST. An obvious one can be found in robotics for “Simultaneous Localization and Mapping” (SLAM) by combining data from different sensors [26]. Every sensor serves as an agent and is source of a piece of evidence. Decisions are based on the linkage of these pieces of evidence by ECR.

Another important application is in combining classifiers as outlined in 2002 by Al-Ani [27]. Elements of a mostly high-dimensional feature space are to be classified into a number of labeled categories. In general, this will require random forest classification or the like. A possible approach via DST will consider the set of labeled categories to represent a frame of discernment. Each classifier (for each feature vector individually) is then transformed into a single piece of evidence by assigning a BBM to all subsets of the appropriate labels. The conjunction of classifiers is again accomplished by linkage of these pieces of evidence using customized ECRs [28].

The described procedure is very close to our approach with the crucial difference, that in our model not the full feature space is mapped to categories, but leaves the option of a feature vector being mapped to an additional category labeled as “undecidable”.

## 2. Materials and Methods

### 2.1. Dempster-Shafer Theory

**Figure 1:**
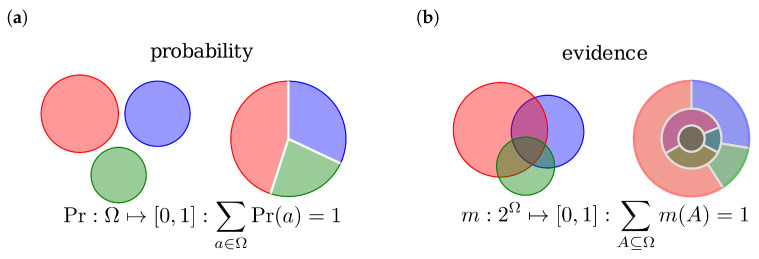
Graphical comparison between probability and evidence: (**a**) a distribution with probability function Pr(a). (**b**) an evidence represented by a mass functions m(A). Note, with three overlapping disks due to the lack of degrees of freedom (5 instead of 6) not all possible constellations can be graphically represented.

Evidence theory by Dempster-Shafer (DST) is based on combining pieces of evidence rather than dealing with probabilities. An evidence can be seen as a generalization of a probability function. The essential difference is, that while in the former the sample space Ω is mapped to probabilities Pr(a),a∈Ω, in DST the power set of the sample space, now called “frame of discernment” (FOD), $P\left(\Omega \right)=2^{\Omega }$ is mapped to masses m(A),A⊆Ω. The mass function m(A) assigns basic belief masses (BBM) to the elements A∈P(Ω) and can be interpreted as degrees of trust in some proposition *A*. Figure 1 shows this for a sample space respectively FOD with the three possible outcomes Blue, Red and Green, thus Ω={B,R,G}.

**Figure 2:**
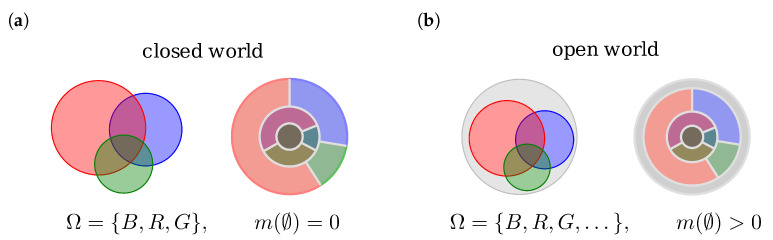
Closed world vs. open world assumption: (**a**) In a closed world no mass is given to the empty set, thus no outcome beyond Ω is possible. (**b**) In an open world a basic belief mass is given to the empty set allowing the ability to consider completely unexpected events to the model (e.g., broken coin) or to deal with data of low quality.

The function m(A):P(Ω)→[0,1] satisfying $\sum_{A\subseteq \Omega }m\left(A\right)=1$ represents an evidence and, in return, every evidence is represented by such a function. Special care has to be taken for the the empty set m(∅) of BBM. If m(∅)=0, an evidence is called normalized. A closed vs. an open FOD are referred to respectively a closed world vs. an open world assumption, see Figure 2.

We currently restrict ourselves to normalized evidence, but we will discuss the origin and opportunities of open world models later in the context of evidence combination rules (ECR) and vague FOD.

For every set S∈P(Ω) the mass function m(A) intrinsically defines two essential quantities of DST, the “Belief” and the “Plausibility” of the set *S*.

(1) $$\begin{array}{ccc} \mathrm{Bel}\left(S\right)=\sum_{A\subseteq S}m\left(A\right) & \; & \mathrm{Pl}\left(S\right)=\sum_{A\cap S\neq \emptyset }m\left(A\right) \end{array}$$

This is why DST is also called the theory of belief functions.

**Figure 3:**
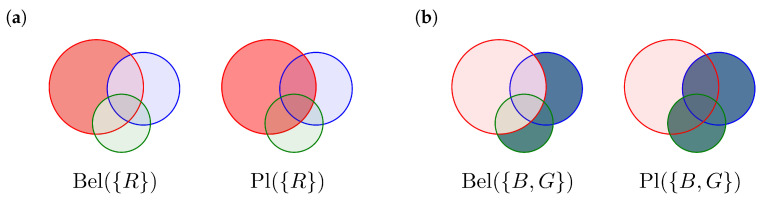
Belief Bel(S) and Plausibility Pl(S) illustrated by overlapping disks. The size of arrays represents basic believe masses. The difference between plausibility and believe Pl(S)\Bel(S) is called uncertainty. (**a**) Bel{R} and Pl{R} of the singleton {R}. (**b**) Bel{B,G} and Pl{B,G} of the set {B,G}.

For a normalized evidence we have Bel(Ω)=Pl(Ω)=1 and Bel(∅)=Pl(∅)=0. This implies that we are sure that the correct answer lies somewhere within Ω (closed world). For better understanding, Figure 3 shows Believe Bel(S) and Plausibility Pl(S) for two elements of P(Ω), namely {R} and {B,G}.

### 2.2. Evidence Combination Rules

One strength of DST is the flexibility in combining pieces of evidence with various ECRs in adjustment of necessities. We will show how to take advantage of this by customizing ECRs, depending on the origin of the data. Put simply, an ECR is a binary operator ⊕ that combines two mass functions $m_{1}\left(A\right)$ and $m_{2}\left(A\right)$ associated with two pieces of evidence with a third mass function m(A), representing a fused evidence.

(2) $$\begin{array}{ccc} m(A) & = & m_{1}\left(A\right)⊕m_{2}\left(A\right) \end{array}$$

Basically, such an operator does not need to fulfill any properties, except ∀A∈P(Ω):m(A)∈[0,1] and $\sum_{A\subseteq \Omega }m\left(A\right)=1$.

Most ECRs are commutative, but only in very rare cases are they associative, not even pseudo-associative in terms of [17]. There is a neutral element $m_{e}\left(A\right)$, called the “vacuous mass function”, satisfying $m\left(A\right)=m_{e}\left(A\right)⊕m\left(A\right)=m\left(A\right)⊕m_{e}\left(A\right)$ with $m_{e}\left(\Omega \right)=1$ and $m_{e}\left(A\right)=0$ for A≠Ω. The evidence associated with $m_{e}\left(A\right)$ is also called “total ignorance”, representing the lack of knowledge. Note that $m\left(A\right)=m\left(A\right)⊕m^{*}\left(A\right)$ does not necessarily imply that $m^{*}\left(A\right)$ is a vacuous mass function. A counterexample is given in [29].

Obviously, an inverse function $m^{-1}\left(A\right)$ with $m\left(A\right)⊗m^{-1}\left(A\right)=m^{-1}\left(A\right)⊗m\left(A\right)=m_{e}\left(A\right)$ for every m(A) does not necessarily exist. This is easy to understand when considering that for a given evidence represented by a mass function m(A) it is unlikely to find more evidence which results in total ignorance. In general, some knowledge brought together with some other knowledge cannot end in knowing nothing. In algebraic terms, the set of all possible m(A) therefore has the structure of an unital magma [30].

An easy way to combine pieces of evidence is by simply multiplying the intersecting mass functions [8].

(3) $$\begin{array}{ccc} m_{1}\left(A\right)⊗m_{2}\left(A\right) & = & \sum_{B\cap C=A}m_{1}\left(B\right)m_{2}\left(C\right) \end{array}$$

**Figure 4:**
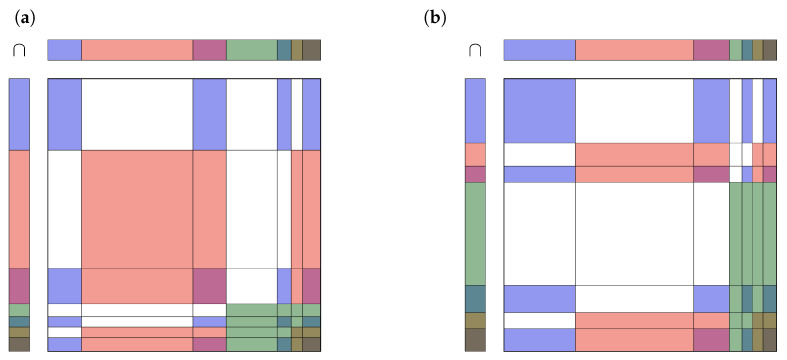
Intersection of basic belief masses of two pieces of evidence. The seven colors in the mosaic plot represent (in order) the seven sets {B}, {R}, {B,R}, {G}, {B,G}, {R,G}, {B,R,G}. The 49 rectangles within the two squares are colored according to the intersect. White represents the empty set *∅*. For a closed world, the white areas must be redistributed among all others. (**a**) rather consent pieces of evidence. (**b**) rather contradicting pieces of evidence.

This ECR is called the “conjunctive” rule [26] and is fully compatible with the open world assumption in the TBM framework. Unfortunately, the resulting mass function $m\left(A\right)=m_{1}\left(A\right)⊗m_{2}\left(A\right)$ is not normalized, so m(∅)≠0. Figure 4 illustrates the Formula (3) for two different cases in mosaic plots, the left one with rather consistent evidence, the right one with rather contradictory evidence (for details see Appendix A).

The 49 rectangles within the two squares are colored in the color of the corresponding intersect, where the white areas are masses for contradicting evidence. Areas of the same color are added. Note that the mosaic plots in Figure 4 can be seen as an operation table for the operator ∩, thus e.g., {R}∩{B,R}={R}, and so on. For a closed world, the white areas must be redistributed among all others.

How to distribute the mass of the empty set m(∅) among all other masses m(A) depends on the needs of the model. If there is a high chance of contradiction between $m_{1}\left(A\right)$ and $m_{2}\left(A\right)$, most of m(∅) will be allocated to m(Ω). Contrarily, if there is a low chance of contradiction, m(∅) will be distributed equally along the singletons m({a}), a∈Ω. As discussed in the introduction, there is a wide range of possible allocations to do so.

For a model to distinguish between hormone receptor statuses it is convenient to use a parameterized family $E={\left\{{⊕}_{\lambda }\right\}}_{\lambda \in [0,1]}$ of ECRs, which is very similar to the one introduced [31], but uses a parameter λ∈[0,1] to customize local requirements. Given two mass functions $m_{1}\left(A\right)$ and $m_{2}\left(A\right)$ we define

(4) $$\begin{array}{ccc} m\left(A\right)=m_{1}\left(A\right){⊕}_{\lambda }m_{2}\left(A\right) & = & \left\{\begin{array}{cc} 0 & A=\emptyset \\ \frac{\sum_{B\cap C=A}m_{1}\left(B\right)m_{2}\left(C\right)}{1-\lambda \sum_{B\cap C=\emptyset }m_{1}\left(B\right)m_{2}\left(C\right)} & A\subset \Omega \\ 1-\sum_{S\subset \Omega }m\left(S\right) & A=\Omega \end{array}\right. \end{array}$$

The parameter λ in Formula (4) provides flexibility to adapt to circumstances. The restriction to λ≤1 is motivated by restricting ourselves to an interpolation type ECR. The value λ>1 would yield an extrapolation type ECR as described in [31].

Dempster’s original ECR [4] is equivalent to setting λ=1. This ECR is associative and commutative. Unfortunately, it turns out that this particular ECR causes significant problems when given pieces of evidence that are rather contradictory [4]. The reason for this is that only the non-contradictory intersect between the two concatenated pieces of evidence is used for the evaluation of the new masses. If this intersect is small, the re-scaling due to normalization blurs out information.

In contrast, Yager [16] distributes all contradicting mass to m(Ω) which is equivalent to setting λ=0. For most applications this approach is too conservative and hinders merging similar evidence to a stronger evidence. However, if pieces of evidence originate from different types of sources this ECR could be very helpful.

Depending on the relation between the agents, different values of λ will be adequate. For pieces of evidence tending to contradict one another, such as combining gene expression with immunohistochemical measurements (IHC), a small value of λ will be favored. For pieces of evidence with low probability of being contradictory, such as combining gene expression from a receptor gene with the co-gene, a greater value of λ will better allow consolidation evidence gained by gene expression. In any case, we should always avoid giving too much weight to any element of P(Ω), especially singletons.

Another major benefit of introducing λ is when combining different receptor statuses to one hormone receptor status. We found that this operation can also be represented by some adequate elements ${⊕}_{\lambda }\in E$. Our data suggests a value of λ≈0.5 as optimum for this task. An illustrated example of evidence linkage and the implications of λ can be found in Appendix A.

## 3. Results

### 3.1. Model Adaptation

For hormone receptor determination the FOD is restricted to two outcomes, hormone receptor positive and hormone receptor negative. We will assume a closed world, thus there are no other possible outcomes than the two elements of Ω.

(5) $$\begin{array}{ccc} \Omega & = & \left\{+,-\right\} \end{array}$$

The simplicity of this model allows us to describe all BBMs by using only two parameters, α and β.

(6) $$\begin{array}{c} m(\{+\})=\alpha \;m(\{-\})=\beta \;m(\{+,-\})=m(\Omega )=1-\alpha -\beta \end{array}$$

The current model involves 6 independent data sources to generate evidence. Four of them originate in gene expression, two in IHC measurements. The gene expression data consists of normalized values for the abundance of estrogen, co-estrogen, progesterone and co-progesterone, where the co-genes are genes closely related to the receptor genes themselves. How to transform gene expression data into BBM given by $\alpha_{\mathrm{expr}}$ and $\beta_{\mathrm{expr}}$ is the subject of our previous papers [32,33].

IHC data originates in the IHC-measurements of estrogen and progesterone receptors. These measurements can be either continuous or discrete (or even missing). How to transform this data into appropriate $\alpha_{\mathrm{ihc}}$ and $\beta_{\mathrm{ihc}}$ is also previously discussed [32,33].

Putting these together into our existing model, the BBM $m_{\mathrm{horm}}$ describing the evidence of the hormone receptor status is calculated as

(7) $$\begin{array}{ccc} m_{\mathrm{horm}} & = & \left(\left(m_{\mathrm{expr}}^{\mathrm{esr}}{⊕}_{1}m_{\mathrm{co}}^{\mathrm{esr}}\right){⊕}_{0}m_{\mathrm{ihc}}^{\mathrm{esr}}\right)⊗\left(\left(m_{\mathrm{expr}}^{\mathrm{pgr}}{⊕}_{1}m_{\mathrm{co}}^{\mathrm{pgr}}\right){⊕}_{0}m_{\mathrm{ihc}}^{\mathrm{pgr}}\right)\\ & = & m^{\mathrm{esr}}⊗m^{\mathrm{pgr}} \end{array}$$

Missing data are represented by the vacuous mass function. On the basis of the Formula (4) ${⊕}_{1}$ stands for Dempster’s ECR and ${⊕}_{0}$ stands for Yager’s ECR respectively. The operator ⊗ does not represent a typical ECR, but a formal procedure reflecting common clinical decision making as given in the below Formula (8).

(8) $$\begin{array}{ccc} \alpha_{\mathrm{horm}} & = & m_{\mathrm{horm}}\left(\left\{+\right\}\right)\\ & = & \left(m^{\mathrm{esr}}⊗m^{\mathrm{pgr}}\right)\left(\left\{+\right\}\right)\\ & = & \max(m^{\mathrm{esr}}\left(\left\{+\right\}\right),m^{\mathrm{pgr}}\left(\left\{+\right\}\right))\\ & = & \max(\alpha_{\mathrm{esr}},\alpha_{\mathrm{pgr}})\\ \beta_{\mathrm{horm}} & = & m_{\mathrm{horm}}\left(\left\{-\right\}\right)\\ & = & \left(m^{\mathrm{esr}}⊗m^{\mathrm{pgr}}\right)\left(\left\{-\right\}\right)\\ & = & \min(m^{\mathrm{esr}}\left(\left\{-\right\}\right),m^{\mathrm{pgr}}\left(\left\{-\right\}\right))\\ & = & \min(\beta_{\mathrm{esr}},\beta_{\mathrm{pgr}}) \end{array}$$

However, this model suffers from a couple of shortcomings. The following list of improvements addresses the problems and provides credible results.

- The operator ⊗, as defined in Formula (8), is not fully compatible with DST. There is always a dependence between the two receptor status. However, DST in its original form requires independent BBMs. This is obviously not the case for estrogen and progesterone receptors. Our suggestion to absorb this correlation is to replace ⊗ by ${⊕}_{0.5}$ giving estrogen and progesterone a balanced contribution to both BBM.
- The operator ${⊕}_{1}$ for combining pieces of evidence coming from gene expression and co-gene expression might be problematic in case of conflicting expression values. In a previous paper [32] we introduced mass limits $\hat{\alpha }$ and $\hat{\beta }$ for the BBMs to tackle this issue. We retain these mass limits, but replace ${⊕}_{1}$ by ${⊕}_{0.9}$ as an additional reinsurance.
- Combining gene expression evidence with IHC evidence, the operator ${⊕}_{0}$ will in case of conflict put too much weight into the mass of ignorance, m(Ω). Therefore we suggest slightly increasing λ and replacing ${⊕}_{0}$ by ${⊕}_{0.1}$. On the lower end of the λ-range, the influence of λ on the ECR is significantly less than on the upper end. As long as there is a profound confidence in the data, particularly in the IHC measurements, replacing ${⊕}_{0}$ by e.g., ${⊕}_{0.3}$ is therefore also an option.
- In the past it turned out that the optimal choice for the co-gene of progesterone is mostly estrogen itself. If so, although $m_{\mathrm{expr}}^{\mathrm{esr}}$ and $m_{\mathrm{co}}^{\mathrm{pgr}}$ are calculated differently and so vary numerically, they are basically generated from the same gene expression data. A preferable assumption in DST is the independence of input data to generate evidence. In contrary to estrogen, progesterone expression data is often diffuse and it might be impossible to find a decent co-gene. This issue can be easily resolved by replacing $m_{\mathrm{co}}^{\mathrm{pgr}}$ with the vacuous mass function. Currently, for the sake of consistency, we stick to the current configuration which uses estrogen as co-gene for progesterone.

Respecting all these issues above we suggest an improved model such as

(9) $$\begin{array}{ccc} m_{\mathrm{horm}} & = & \left(\left(m_{\mathrm{expr}}^{\mathrm{esr}}{⊕}_{0.9}m_{\mathrm{co}}^{\mathrm{esr}}\right){⊕}_{0.1}m_{\mathrm{ihc}}^{\mathrm{esr}}\right){⊕}_{0.5}\left(\left(m_{\mathrm{expr}}^{\mathrm{pgr}}{⊕}_{0.9}m_{\mathrm{co}}^{\mathrm{pgr}}\right){⊕}_{0.1}m_{\mathrm{ihc}}^{\mathrm{pgr}}\right) \end{array}$$

**Figure 5:**
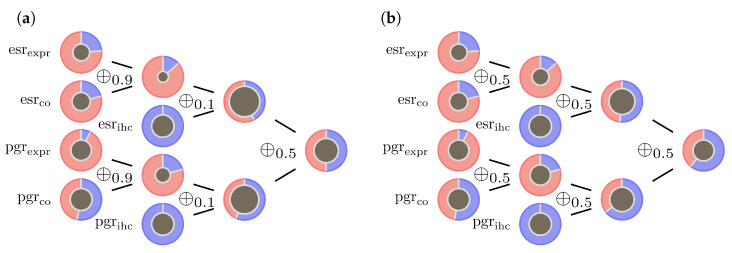
Contradictory data inducing undecidable outcome: (**a**) model (9) illustrated by a sample with indecisive outcome (sample id = 881). Both IHC measurements ${\mathrm{esr}}_{\mathrm{ihc}}$ and ${\mathrm{pgr}}_{\mathrm{ihc}}$ are receptor negative, but three out of 4 pieces of evidence based on gene expression indicate a receptor positive status. Red areas represent masses α for positive hormone status, blue areas represent masses β for negative hormone status, centers represent masses θ=1−α−β for Ω={+,−}. (**b**) choosing inappropriate λ for the ECRs results in dubious prognosis.

**Figure 6:**
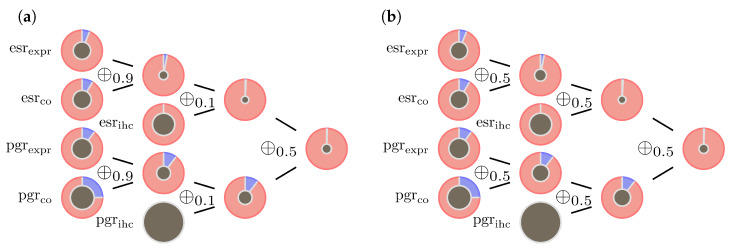
Consistent data inducing reliable outcome: (**a**) model (9) illustrated by a sample with very clear outcome (sample id = 1980). (**b**) When evidence is highly consistent, the parameter λ has practically no influence on the results.

The operators ${⊕}_{0.1}$ and ${⊕}_{0.9}$ are small derivations from to the original model and mainly serve to increase prediction stability in the case as described in [5]. Graphic examples are shown in Figure 5 and Figure 6.

A further detailed explanation is required for the shift from ⊗ to ${⊕}_{0.5}$. For a sample to be receptor positive, only one of the two receptors (estrogen OR progesterone) needs to be positive while for being negative both receptors (estrogen AND progesterone) have to be negative. Therefore, the operator ⊗ as given in Formula (8) will fail and produce a misleading shift towards hormone receptor positive. If one of the two receptors has medium evidence for being positive and the other receptor has strong evidence for being negative, the operator ⊗ will still result in an evidence favoring a positive outcome hormone receptor.

Moreover, there is a strong connection between the two receptor genes. A progesterone positive sample will almost always be estrogen positive while an estrogen negative sample is very likely to be progesterone negative. However, the approach given in Formula (8) is based on the assumption of almost independent receptors.

Combining receptor evidence with ${⊕}_{0.5}$ will, on the other hand, fix the above issues. In both cases it is still very likely that the hormone receptor status concluded from the evidence will be classified as “undecidable”, but in case of misclassification the probability of erroneously positive classified samples will be reduced by a large amount. This is in line with clinical demands.

### 3.2. Examples

The data set for the following results consists of 2559 freely available breast cancer samples from the Gene Expression Omnibus [34]. For each sample, at least one IHC measurement of a hormone receptor was performed as part of the respective study. Details can be found in Appendix B.

In the first example (sample id 881 from the data set), gene expression data and IHC measurements are contradicting each other. In addition, gene expression of progesterone is not very accurate, and can be seen from the differing measurements. This leads to a final very diffuse evidence, and therefore no decision can reliably be made.

Figure 5a shows the evolution of evidence for this particular sample. Figure 5b shows the importance of choosing the right λ for the ECRs. The example shows that merging gene expression evidence with unclear IHC evidence can result in a dubious prognosis when choosing a too large λ.

In the second example (sample id 1980 from the data set), there is strong conformity in the data. Although one IHC measurement is missing, pieces of evidence accumulate to a strong belief in hormone receptor positive, see left panel in Figure 6. The right panel in Figure 6 demonstrates that in case of consistent evidence the influence of the parameter λ can be neglected.

**Figure 7:**
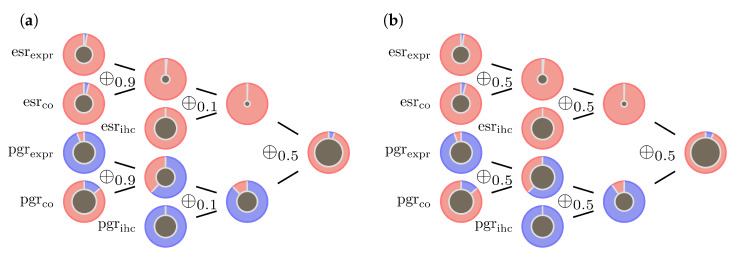
Contradicting data inducing uncertainty: (**a**) model (9) illustrated by a sample with very contradictory input data (sample id = 2365). (**b**) even when setting all λ=0.5 in the ECRs no conclusive evidence is generated. Nevertheless, the influence of λ can change case by case.

The last example (sample id 2365 from the data set) is a very contradictory example concerning the data at hand. Large amounts of the final mass are distributed to lack of knowledge, which can be seen from the large central circles in Figure 7. The influence of λ can change from case to case.

### 3.3. Analysis

**Figure 8:**
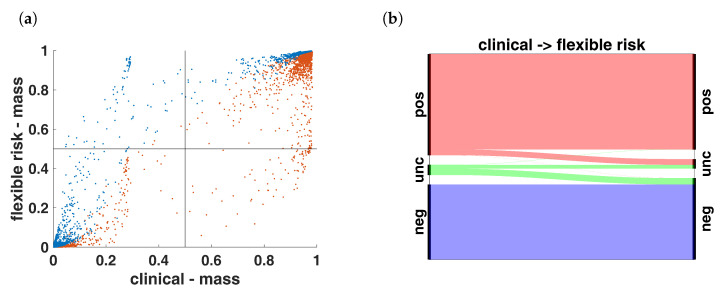
Illustration of the shift towards hormone receptor negative outcome by an improved linkage between hormone receptors (Formula (9)): (**a**) red dots are weights α for receptor positive, blue dots are weights β for receptor negative. (**b**) incorrect favoring of positive hormone receptor status has been revised by using ${⊕}_{0.5}$ instead of ⊗.

As can be seen in Table 1a and Figure 8, the switch from our previous approach (Formula (7)) to an improved linkage between the two hormone receptors (Formula (9)) entails a shift towards receptor negative. This is reflected by 86 samples clinically classified as “uncertain”, now being classified as “receptor negative” and 78 samples clinically classified as “receptor positive”, now being classified as “uncertain”. This shift can be quantified by a Cohen’s κ=0.877. Using a constant λ for ECRs instead of (9) only has an influence on numerically problematic samples, as can be seen in Table 1b.

The left panel of Figure 8 shows the change in the α (red dots) and β (blue dots), while the right panel illustrates Table 1 in an alluvial diagram.

### 3.4. Decision Making

We will not change our strategy for decision making as proposed in our previous work [32,33]. This means, we consider an outcome *A* as “true” if the belief in it has more mass than the plausibility of its complement $A^{\prime }$. Let T⊆P(Ω) be the subset of all “true” elements of P(Ω).

$$\begin{array}{ccc} A\in T & ⟺ & \mathrm{Bel}\left(A\right)>\mathrm{Pl}\left(A^{\prime }\right)\;A^{\prime }=\Omega \backslash A \end{array}$$

In this very simplified case with Ω={+,−} it reduces to

$$\begin{array}{ccc} \{+\}\in T⟺\alpha >0.5 & \; & \{-\}\in T⟺\beta >0.5 \end{array}$$

Note that Ω∈T will clearly always hold under the closed world assumption.

## 4. Discussion

### 4.1. Quality of Data

In DST, the dogma “a (machine learning) model is only as good as the data it is fed” can be understood from a different perspective. This guiding principle is still valid, but lack of data quality can be coped with in the BBMs and ECRs by adequate parametrization. Here DST offers additional flexibility.

In our model with only two possible outcomes this is simple. The best example is the modeling of the BBM for the IHC status. The less confidence there is in the data, the more mass is assigned to subsets of Ω with more than one element, i.e., blurred decisions. With increasing confidence in the IHC measurements, the corresponding singletons (i.e., crisp decisions) are more highly valued.

### 4.2. From Data to Evidence

This issue was the subject of our earlier papers [32,33] and we will therefore only briefly discuss it. Gene expression values are converted into BBM using logistic regression. In addition, two mass limits $\hat{\alpha }$ and $\hat{\beta }$ are introduced for the following purposes.

The most important is to consider the possibility of erroneous gene expression values by keeping masses significantly smaller than 1. A welcome side effect is to avoid some rare numerically problematic cases.

The conversion of IHC measurements into BBM is again realized as described in [32,33]. We assume that about 85% of the IHC measurements are correct.

### 4.3. The Functionality of λ in ${⊕}_{\lambda }$

We introduced the parameter λ to specifically adapt decision strategy to the properties of data and its origin from which evidence is to be generated. The more data sources differ in nature, the smaller λ should be chosen. This prevents too much mass accumulating in the singletons when mixing conflicting evidence. We call this strategy a conservative ECR.

On the other hand, if the data sources are homogeneous, a large λ can be chosen. We call this case a risky ECR. In the case of a risky ECR, care must be taken to ensure that contradictory singletons do not enter simultaneously with masses close to 1. In our model, this case is prevented by the mass limits $\hat{\alpha }$ and $\hat{\beta }$.

Theoretically, it would also be possible to choose λ>1 as suggested [31]. That would correspond to an extrapolation in the sense that two consistent pieces of evidence not only increase certainty but also amplify each other to something stronger than the sum of them. However, our focus is in finding possible contradictions in data and therefore we see no point in merging evidence for hormone receptor status determination with λ>1.

**Figure 9:**
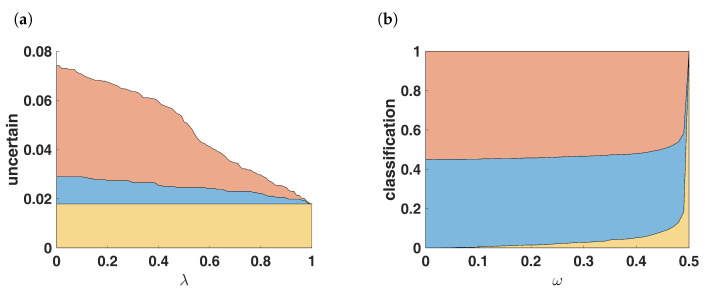
Uncertainty vs. flexible risk. (**a**) Increasing risk decreases uncertainty: The number of uncertain samples depends on the parameter λ in combining the hormone receptors with $m^{\mathrm{esr}}⊗m^{\mathrm{pgr}}$. The yellow area shows 45 out of 2519 samples (1.8%) which will always be uncertain, independent of the choice of λ. The red area changes from uncertain to receptor positive with increasing λ, the blue area changes into receptor negative. (**b**) fixed mass of ignorance: number of classifications by fixing the weight m(Ω)=ω as described by Formula (11).

There is even more potential in the variation of λ when combining the two hormone receptors, estrogen and progesterone. Our suggestion (Formula (9)) is choosing λ=0.5. By varying λ in the linkage between the two hormone receptors, the amount of unclassified samples can be regulated conveniently. This is illustrated in Figure 9.

### 4.4. Training of λ in ${⊕}_{\lambda }$ on Real Data

The parameter λ is currently set according to intuitive arguments rather than strict mathematical rules. It would be interesting to investigate the existence of an algorithm to calculate λ depending on arbitrary training data and to develop a mechanism that suggests an optimal choice.

Due to a lack of clean training data of sufficient high quality we have done simulations to train λ appropriately. It turned out that this task is far from trivial and needs further investigation.

### 4.5. Enhanced Evidence Combination Rules

There are many ECRs available and existing ones are constantly being developed further. However, none of these developments could state with certainty or explain conclusively which ECR is beneficial for which application. Therefore we proposed to set the parameter λ according to expert knowledge on the nature of the data.

In any case, the parameterized ECR introduced in this way covers a very wide range of possible combinations of evidence. Unfortunately, it is difficult to assess whether certain additional mathematical requirements for an ECR, such as commutativity, (pseudo-) associativity, idem-potency, invertibility or other characteristics of binary operators could provide additional value.

### 4.6. An Evidence Combination Rule with Constant Ignorance

The larger the expected contradiction between the two BBMs, the smaller one will usually choose λ. On the other hand, if expected contradictions are small, increasing risk may be taken by choosing a larger λ. The question arises as to why one must choose λ at all and not take a λ adapted according to some formula. For example, the reciprocal value of the total mass could be used as the setting point:

(10) $$\begin{array}{ccc} \lambda & \propto & \frac{1}{\sum_{B\cap C=\emptyset }m_{1}\left(B\right)m_{2}\left(C\right)} \end{array}$$

The net effect of this strategy would be to reduce the variability of m(Ω) within the samples.

This approach can be pursued particularly elegantly if one assumes the resulting mass of total ignorance as an a priori constant, i.e., m(Ω)=ω. The corresponding ECR would then read

(11) $$\begin{array}{ccc} m\left(A\right)=m_{1}\left(A\right){⊕}_{\lambda }m_{2}\left(A\right) & = & \left\{\begin{array}{cc} 0 & A=\emptyset \\ \frac{\left(1-\omega \right)\sum_{B\cap C=A}m_{1}\left(B\right)m_{2}\left(C\right)}{1-m_{1}\left(\Omega \right)m_{2}\left(\Omega \right)-\sum_{B\cap C=\emptyset }m_{1}\left(B\right)m_{2}\left(C\right)} & A\subset \Omega \\ \omega & A=\Omega \end{array}\right. \end{array}$$

For the special case Ω={+,−}, as valid for hormone receptor determination, this is directly leading to α+β=1−ω, with ω representing the basic belief mass of total ignorance.

### 4.7. Modified Frame of Discernment

Our FOD consists of only two outcomes, “positive” and “negative”, i.e., Ω={+,−}. In practice, however, the hormone receptor status is not solely responsible for the therapeutic decisions on therapy. There are different types of receptor-positive patients, and not all will respond equally well to hormone therapy. Therefore expanding the presented model at a later time is inevitable. There are two possible approaches to do so:

The first is a refined model. Here, the refinement lies in subdividing “+” further into $\left\{+\right\}=\left\{{+}_{1},{+}_{0}\right\}$, i.e., $\Omega =\{{+}_{1},{+}_{0},-\}$, which can easily be arranged with part of the clinical data, since the ESR receptor status is often given as a (quasi-)continuous parameter.

The second is to adapt the “open world assumption”. There are patients for whom it is basically impossible to make a serious choice for the most suitable treatment method based on the receptor status–even if it is measured precisely and reliably. The outcome for such patients is therefore not covered by the FOD. Such a model can be implemented by allowing a strictly positive BBM of the empty set, ergo m(∅)>0.

### 4.8. Risk Function for Decision Making

Finally, another open point is the need for a risk function. Wrong decisions regarding therapy are not symmetrical. Adjuvant chemotherapy is often vital, even if only hormone therapy is applied. Some preliminary investigations have already been carried out [25], but considering the specific case, it is still an open field of research. Creating such risk functions is a heavily investigated topic and we will come back to it in a succeeding paper.

## 5. Conclusions

Dempster–Shafer theory of belief functions represents a generalization of Bayes’ probabilities. It provides a powerful framework to proactively involve the outcome “uncertain” in case of insufficient data availability to make confident decisions. Instead of probabilities pieces of evidence, represented by basic belief masses, are concatenated by evidence combination rules.

In this paper, we presented a manner to parameterize evidence combination rules to adjust models to the nature of the incoming measurements. Data with high potential to be contradictory (like gene expression and immunohistochemical measurements) is linked in a more conservative manner than data which is more likely expected to be in consent. Thus, evidence theory avoids several well-known problems with decisions based on conventional statistics.

As a major advancement, our work introduces flexible evidence-combination-rules offering the potential of adaptable risk. Changing the parameters for concatenating pieces of evidence (respectively data) alters the probability for a sample to be classified as “well-defined” or as “uncertain”. This is especially helpful to adapt the Dempster-Shafer algorithm to possible different types of risks and directly related possibilities of a treatment decision. Examples are possible over- and under-treatment of particular patients.

To illustrate the strength of evidence theory we used a case study of hormone receptor status determined for breast cancer samples. As a key outcome we estimate that slightly too many patients have been classified as hormone receptor positive by conventional clinical-decision-making in comparison to our approach. We do not advocate overruling clinical decisions, but rather flagging questionable samples as “uncertain” and suggesting further investigations for these particular patients.

Drawing on flexible evidence combination rules in our approach we see great potential for the advancement in personalized medicine.

## Figures and Tables

**Table 1:** Clinical decision making vs. flexible risk: (**a**) change from clinical decision making to Formula (9), κ=0.877, there is a trend towards receptor negative (upper triangular matrix). (**b**) an influence of λ is only given for numerically problematic samples, κ=0.966.

(**a**)				
		flexible risk		
		pos	unc	neg
clinical	pos	1287	78	1
	unc	3	51	86
	neg	0	0	1013
(**b**)				
		λ=0.5 constant		
		pos	unc	neg
flexible risk	pos	1268	22	0
	unc	14	107	8
	neg	0	2	1098

**Figure A1:**
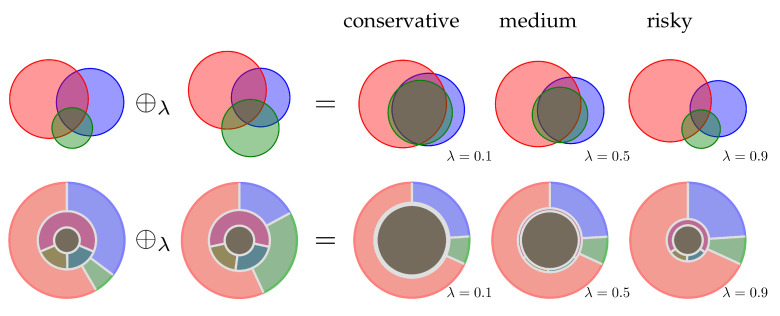
Adding two rather consistent pieces of evidence with Formula (4). A larger value of λ (less conservative) seems to be preferable. Masses for ambiguous outcomes such as {R,B} almost vanish. The first and the second rows show different graphical representations of the same situation.

**Figure A2:**
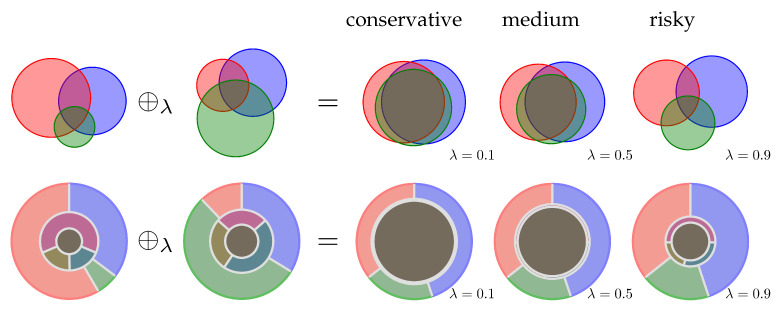
Adding two rather contradictory pieces of evidence with Formula (4). A smaller value of λ (more conservative) seems preferable staying on the safe side. Obviously none of the three possible outcomes receives the necessary support to represent a good choice. Contradicting masses should therefore be mostly allocated to total ignorance, m(7)=m(Ω) (compare Formulas (A1) and (A2)), displayed by the central grey disk.

## Data Availability

All data were downloaded from Gene Expression Omnibus [34].

## Abbreviations

The following abbreviations are used in this manuscript:

BBM	basic belief masses, same as basic belief assignment (BBA)
BC	breast cancer
DSmT	Dezert-Smarandache theory
DST	Dempster-Shafer theory of evidence
ECR	evidence combination rule
ESR	estrogen
FOD	frame of discernment
GEO	Gene Expression Omnibus
IHC	immunohistochemistry
PCR	proportional conflict redistribution
PGR	progesterone
SLAM	simultaneous localization and mapping
TBM	transferable belief model

## Appendix A. Examples of Combining Pieces of Evidence

For convenience we denote every element S∈P(Ω) by an integer a(S). Let $\Omega =\{x_{0},x_{1},\ldots x_{n-1}\}$ and S⊆Ω. We define

(A1) $$\begin{array}{ccc} a(S) & = & \sum_{k=0}^{n-1}\chi_{S}\left(x_{k}\right)2^{k} \end{array}$$

with indicator function

(A2) $$\begin{array}{ccc} \chi_{S}\left(y\right) & = & \;\left\{\begin{array}{cc} 1 & \mathrm{if}\;\;y\in S\\ 0 & \mathrm{if}\;\;y\notin S \end{array}\right. \end{array}$$

and identify m(S)≡m(a(S))≡m(a). So m(5) would be the mass of $\left\{x_{0},x_{2}\right\}$.

For illustration of a general case we consider three mutually exclusive outcomes (e.g., treatments) Ω={B,R,G} and two pieces of evidence from agents $E_{1}$ and $E_{2}$ with mass functions $m_{i}\left(a\right):A\to \left[0,1\right]$, A={1,2,…,7}, i∈{1,2} in the notation above. Note that for this demonstration example we assume a closed FOD and therefore do not explicitly write down $m_{1}\left(0\right)=m_{2}\left(0\right)=0$ hereinafter.

### Appendix A.1. Two Rather Consistent Agents

This case often occurs when data from similar sources are to be linked. This could be multiple measurements of the same parameter within a short time span, but also correlated genes from one gene expression chip.

The example in Figure A1 gives two agents strongly agreeing in Red. While the first one considers Blue as an alternative, the second one’s first alternative is the preferable Green.

$$\begin{array}{ccc} m_{1}\left(A\right) & = & \{0.262,0.434,0.130,0.047,0.038,0.039,0.050\}\\ m_{2}\left(A\right) & = & \{0.123,0.409,0.122,0.187,0.051,0.042,0.066\}\\ \left(m_{1}{⊕}_{\lambda }m_{2}\right)\left(A\right) & = & \left\{\begin{array}{cc} \{0.138,0.393,0.032,0.045,0.007,0.007,0.378\} & \lambda =0.1\\ \{0.166,0.471,0.038,0.054,0.009,0.008,0.254\} & \lambda =0.5\\ \{0.207,0.589,0.048,0.068,0.011,0.010,0.067\} & \lambda =0.9 \end{array}\right. \end{array}$$

### Appendix A.2. Two Rather Contradictory Agents

When different types of data are to be linked, this case is likely to occur. In our example this is data from immunohistochemistry with data from gene expression. It will occur more often when evidence based on different opinions or measurement methods are to be linked.

The example in Figure A2 shows two agents, where the first one strongly believes in Red, while the second one gives most mass to Blue or Green.

$$\begin{array}{ccc} m_{1}\left(A\right) & = & \{0.262,0.434,0.130,0.047,0.038,0.039,0.050\}\\ m_{3}\left(A\right) & = & \{0.236,0.085,0.059,0.378,0.100,0.059,0.083\}\\ \left(m_{1}{⊕}_{\lambda }m_{3}\right)\left(A\right) & = & \left\{\begin{array}{cc} \{0.203,0.161,0.023,0.088,0.013,0.009,0.504\} & \lambda =0.1\\ \{0.260,0.207,0.029,0.113,0.016,0.012,0.363\} & \lambda =0.5\\ \{0.365,0.290,0.041,0.158,0.023,0.016,0.108\} & \lambda =0.9 \end{array}\right. \end{array}$$

## Appendix B. Data Description

The data used for this study are identical to the data set used in our previous publications and is described in detail in [32]. It consists of 3753 samples from 38 studies downloaded from the Gene Expression Omnibus (GEO) [34], including clinical parameters. Of these, 2559 samples were finally selected in which HER2 status could be determined to be negative with reasonable confidence.

Clinical parameters and, in particular, immunohistochemical measurements were obtained either from the GEO database metadata directly or from the associated publications. All input parameters for our models were also subjected to a double plausibility check. Removal of non-biological batch effects in the gene expression data was done as part of a normalization process that included all studies simultaneously.
